# Vernix Caseosa Peritonitis: A Scoping Review

**DOI:** 10.3390/medicina61101786

**Published:** 2025-10-03

**Authors:** Goran Augustin, Mislav Herman, Zrinka Hrgović, Ante Krešo, Jure Krstulović

**Affiliations:** 1 Department of Surgery, University Hospital Centre Zagreb, 10000 Zagreb, Croatia; 2 Department of Gynaecology and Obstetrics, University Hospital Centre Zagreb, 10000 Zagreb, Croatia; mislav.herman@kbc-zagreb.hr; 3 School of Medicine, University of Zagreb, 10000 Zagreb, Croatia; 4 School of Medicine, University of Split, 21000 Split, Croatia; zrinka.hrgovic@mefst.hr (Z.H.); jkrstulovic@kbsplit.hr (J.K.); 5 Department of Family Medicine, Split-Dalmatia Health Center, 21000 Split, Croatia; 6 Department of Ophthalmology, University Hospital of Split, 21000 Split, Croatia; ante.kreso@mefst.hr; 7 Department of Surgery, University Hospital of Split, 21000 Split, Croatia

**Keywords:** vernix caseosa peritonitis, diagnosis, treatment, corticosteroids, outcomes, systematic review

## Abstract

*Background and Objectives*: Vernix caseosa peritonitis (VCP) is rare. Nonspecific symptoms of acute abdomen during early puerperium make preoperative diagnosis of VCP challenging. We aimed to identify risk factors, early diagnosis and treatment options, and the association between the timing and severity of VCP diagnosis and maternal outcomes. *Materials and Methods*: We searched PubMed, PubMed Central, and Google Scholar. Articles were analyzed according to the PRISMA guidelines. The search items included: ‘vernix caseosa peritonitis, ‘vernix caseosa granuloma, ‘maternal meconium peritonitis’, ‘maternal meconium granuloma’, ‘vernix caseosa’, ‘peritonitis’, ‘pregnancy’, ‘puerperium’, ‘postpartum’, and ‘gravid’. Additional studies were extracted by reviewing the reference lists of retrieved studies. Demographic, clinical, obstetric, diagnostic, and treatment parameters, and outcomes were collected. *Results*: Out of 55 published VCP case reports, 46 were available. Most involved term pregnancies (84.8%) and were delivered by Cesarean section (CS) (87%), with no difference in parity distribution (χ^2^(2) = 1.1875, *p* = 0.5523) or fetal sex (m: f = 53.3%: 46.7%). Common symptoms included abdominal pain and fever over 38 °C, while dyspnea or tachypnea was unexpectedly frequent (23.9%/15.2%). The interval from delivery to surgery ranged from 4 to 13 days (average 8 days), with no difference between CS and vaginal deliveries. Preoperative VCP was diagnosed in only 4.3% of cases, and intraoperative diagnosis occurred in 60.9%. Intraoperative microbiology and histopathology (vernix components) were positive in 6.5% and 89.1%, respectively. The birth weight was normal (3656 ± 509 g), with no maternal or neonatal deaths. *Conclusions*: VCP primarily develops in term pregnancies delivered by CS, without other risk factors. Despite extremely low preoperative and unexpectedly low intraoperative diagnosis and treatment delay of several days, there is no maternal or fetal mortality. The time to symptom onset is similar between women who delivered vaginally and those who had a CS. All women with VCP after vaginal delivery had previous deliveries. Abdominal pain with a fever over 38 °C and dyspnea or tachypnea in the early puerperium suggests VCP. Surgical lavage is the primary treatment, while corticosteroids have been reported to be beneficial in several cases, and antibiotics seem to have a limited role.

## 1. Introduction

Vernix caseosa peritonitis (VCP) is an uncommon but clinically significant postpartum complication, particularly after Cesarean sections (CS) [1]. This condition involves an acute inflammatory response caused by the spillage of vernix caseosa—a white, cheese-like substance produced by fetal sebaceous glands—into the maternal peritoneal cavity [2]. Although vernix caseosa provides protective functions for the newborn, such as moisturizing and antimicrobial effects during fetal development, its presence in the maternal peritoneum may trigger (sub)acute granulomatous peritonitis, which can develop several days to weeks after delivery [3,4].

The literature documents a limited number of cases, with estimates indicating approximately 34 reported cases of VCP since 1976 [1]. We identified 55 published cases, of which 46 were available for analysis. CS is a primary risk factor, mainly due to potential leaks of amniotic fluid containing vernix caseosa into the maternal peritoneal cavity [5]. However, cases following vaginal births have also been recorded (13%), showing that VCP should be considered in all childbirth situations, not just CS [6,7]. VCP has various stages of severity. This complexity underscores the need for increased awareness and the identification of signs related to this unique form of peritonitis, as early diagnosis and treatment can prevent significant maternal morbidity [8,9].

Research shows that pathology may involve a foreign body-type granulomatous response, with initial symptoms often mistaken for more common postoperative complications [10]. Typical symptoms such as acute abdominal pain, fever, and signs of sepsis usually occur 3 to 35 days after delivery [5,9]. The imaging findings vary, and the radiological features are not well described [11]. Treatment most often involves diagnostic laparoscopy or laparotomy, followed by peritoneal lavage, often with additional therapies like antibiotics and, in some cases, corticosteroids [11,12].

In conclusion, VCP is an essential concern in obstetric care, requiring clinical awareness and prompt action in response to post-delivery abdominal pain and fever. Better understanding of this condition could significantly enhance maternal outcomes after CS, aligning clinical practices with new trends in postpartum care.

## 2. Materials and Methods

We searched PubMed, PubMed Central, and Google Scholar and analyzed the collected case reports and case series according to the Preferred Reporting Items for Systematic reviews and Meta-Analyses extension for Scoping Reviews (PRISMA-ScR) Checklist. The search terms included: ‘vernix caseosa peritonitis’, ‘vernix caseosa granuloma’, ‘maternal meconium peritonitis’, ‘maternal meconium granuloma’, ‘vernix caseosa’, ‘peritonitis’, ‘pregnancy’, ‘puerperium’, ‘postpartum’, and ‘gravid’. Additional studies were identified by reviewing reference lists of retrieved studies. We included all available full-text cases and case series. We excluded cases with duplicated or overlapping data, insufficient information, patients with postoperative peritonitis, meconium peritonitis, other surgical postpartum emergencies, and patients who were not pregnant or in the puerperium. The search was limited to human studies published up to May 2025, with no restrictions on language or country. Non-English articles were translated with the assistance of medical scientists fluent in those languages.

Our goal was to summarize all reported VCP cases and identify predictive findings for early diagnosis and treatment. Our second objective was to find an association between diagnosis, treatment, and maternal/neonatal outcomes. The study is exempt from ethics approval because we used data from published cases.

### 2.1. Data Extraction

Two authors independently reviewed the extracted data from the included articles. Pre-defined criteria were established to ensure the maximum reliability of the collected data.

Obstetric data included maternal age, gestational age, parity, gravidity, number of deliveries, number of miscarriages, and previous deliveries—vaginal or cesarean. Clinical and patient characteristic data encompassed previous abdominal surgeries, medical history, and indications for previous CS. Diagnostic data comprised symptoms, signs, and radiology findings. Other parameters collected were differential diagnosis, outcomes for mother and child, type of delivery, type of surgery performed, time between delivery and surgery or vice versa, laboratory results, findings from abdominal paracentesis and uterus/cervix swab, intra-abdominal access, intraoperative and postoperative diagnoses, intraoperative pathology, postoperative course, therapy, and recurrent symptoms.

### 2.2. Quality Assessment

The methodological quality of included studies was assessed using the Joanna Briggs Institute (JBI) Critical Appraisal Tools for case reports and case series. The evaluation focused on eight criteria for case reports. Each criterion was scored as “yes,” “no,” “unclear,” or “not applicable.” Studies were rated as “good” if they met ≥75% of the criteria, “fair” if they met 50–74%, and “poor” if they met <50%. Detailed quality assessments for each study are provided in Supplementary Table S1.

### 2.3. Statistical Analysis

Statistical analyses were conducted in R (version 4.3.2; R Foundation for Statistical Computing, Vienna, Austria) after importing the extracted variables into Microsoft Excel 2021. Complete data were unavailable for nine of the 55 published case reports, so these studies were excluded from all analyses; the remaining 46 reports constituted the dataset for analysis.

Categorical data are presented as absolute numbers with corresponding percentages. Continuous variables were tested for normality using the Shapiro–Wilk test and visual assessments of histograms and Q-Q plots. Measures that are approximately normally distributed (e.g., maternal age) are shown as mean ± standard deviation (SD) and range, while skewed measures (e.g., operative time intervals, C-reactive protein) are reported as median and interquartile range (IQR); minimum–maximum values are included when they provide clinical relevance. Differences between groups in nominal variables (e.g., parity categories) were analyzed using Pearson’s χ^2^ test; when any expected cell count was less than 5, Fisher’s exact test was used instead. Due to the descriptive, single-cohort design of the dataset, no multivariable modeling or adjustment for multiple comparisons was performed. All tests were two-sided, and a *p*-value less than 0.05 was considered statistically significant.

## 3. Results

**Figure 1:**
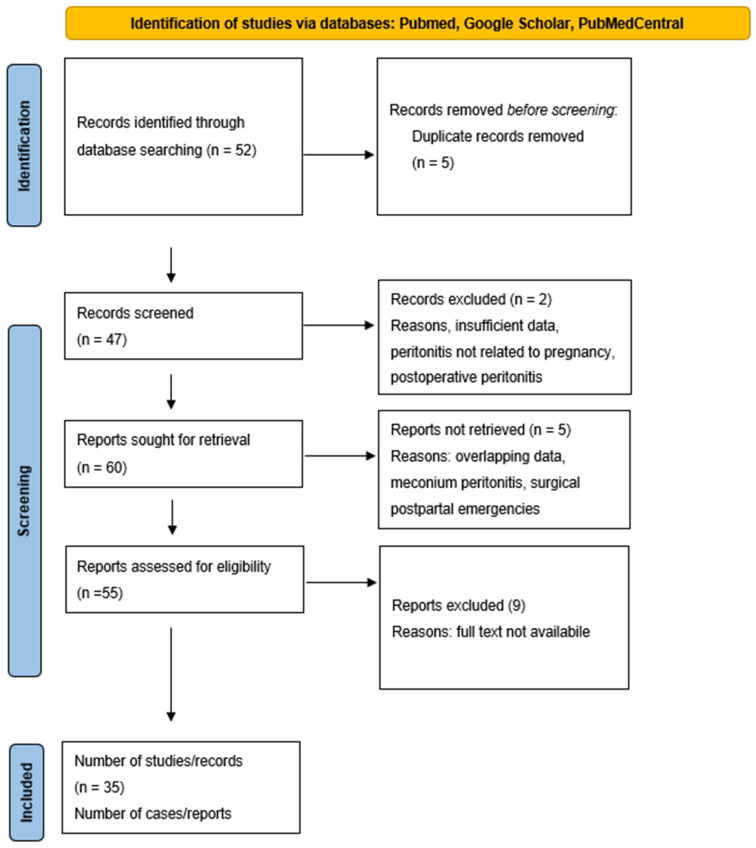
PRISMA flow diagram for vernix caseosa peritonitis.

Data were available for 46 of the 55 published cases of VCP (Figure 1).

The information from individual case reports is summarized in Supplementary Table S2. In this cohort, the mean maternal age was 29 ± 6 years (range, 17–43), and most pregnancies were carried to term (84.8%). Most women delivered by CS (87%). There was no statistically significant difference in parity distribution among nullipara, primipara, and multipara (χ^2^(2) = 1.1875, *p* = 0.5523). Further baseline characteristics are summarized in Table 1.

Six women delivered vaginally (13% of all cases)—two with unknown prior labor history, one with a previous CS, and two with prior vaginal deliveries. One woman, whose first delivery was by CS and second by vaginal birth, delivered vaginally with vacuum extraction. In all six cases, symptoms or signs that could implicate high rupture of membranes were not described. There was no statistically significant difference in the time to symptom onset between women who delivered vaginally and those who underwent CS (Mann–Whitney U = 70, *p* = 0.14).

Among the 40 cases analyzed, the most common indications for CS were failure to progress in labor (27.5%) and breech presentation (20.0%). Elective procedures or cases without a documented indication accounted for 22.5%. Mechanical dystocia was observed in 12.5% of cases, followed by fetal compromise (10.0%), cord prolapse (5.0%), and uterine rupture (2.5%). Preterm rupture of membranes occurred in 7 out of 46 cases (15.2%). Fetal sex was recorded in fewer than a third of cases, equally distributed (m: f = 53.3%: 46.7%). The birthweight was within the normal range (3656 ± 509 g). Adequate wound healing after CS was reported in 20%; no data for the remaining cases (Table 2). In 95% of cases, the post-cesarean course was uneventful. Therefore, it can be assumed that all CS incisions healed uneventfully.

**Figure 2:**
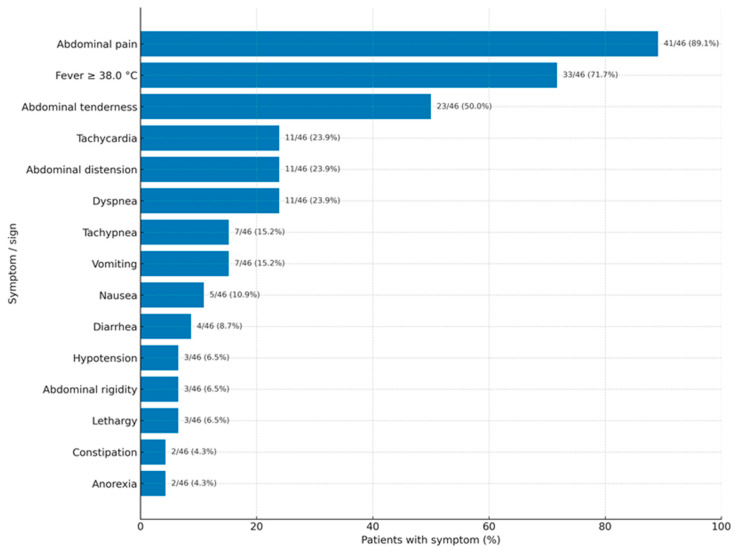
Frequency of symptoms and signs in vernix caseosa peritonitis.

As shown in Figure 2, acute abdominal pain was the main feature of VCP, with fever often occurring alongside. Interestingly, a large number of patients also presented with dyspnea/tachypnea (Figure 2).

Abdominal–pelvic CT was the primary investigation and was usually diagnostic, while ultrasound was used less often and only agreed with CT findings in a minority of cases. Abdominal MRI and chest CT were rarely performed but generally provided supportive information when performed. Plain radiographs and vaginal examinations contributed minimal diagnostic value (Table 3). Notably, vaginal examination is expected to be routinely conducted due to severe postdelivery presentation, but it was documented in only 10.9% of cases. Concordant imaging findings were considered suspicious for vernix caseosa peritonitis, with CT most often showing peritoneal thickening and enhancement, inflammatory fat stranding, free peritoneal fluid, and intraperitoneal heterogeneous material with soft-tissue or fat attenuation. Ultrasound was only concordant in a few cases, identifying free fluid or echogenic intraperitoneal material, while MRI occasionally revealed peritoneal thickening and fluid collections.

Among the 46 patients included in the analysis, the preoperative differential diagnoses varied (Table 4). The most common was acute appendicitis, documented in 7 (15.2%), followed by peritonitis in 5 (10.9%) cases. VCP was initially suspected in only 2 patients (4.3%). Other less frequent preoperative diagnoses included cholecystitis (2 cases; 4.3%), gastroenteritis, visceral injury, endometritis, intraperitoneal abscess, adnexal torsion, myofibroblastic tumor, and ruptured uterine scar—each reported in 1 patient (2.2%). In 23 cases (50%), the preoperative diagnosis was either unknown or not recorded.

In contrast, intraoperative diagnoses (Table 4) showed VCP in 28 of 46 cases (60.9%), making it the most common diagnosis. Acute appendicitis was confirmed in 6 patients (13.0%), and peritonitis of other or unspecified origin in 4 cases (8.7%). Other findings included foreign body granuloma and bowel perforation, each identified in 1 patient (2.2%). In 6 cases (13.0%), no conclusive diagnosis was recorded.

These findings highlight the diagnostic inaccuracy of VCP, which is frequently underrecognized preoperatively and often mimics more common intra-abdominal conditions.

Laparotomy was the primary surgical method, while laparoscopy was less frequently used, and only 6.5% avoided surgery (Table 5). Most procedures were therapeutic rather than purely diagnostic. Surgery was usually performed about a week after delivery, although it was often done immediately after hospital admission.

The surgical field typically contained white cheesy exudates, observed in 19 out of 46 cases (41.3%). Adhesions were present in 15 cases (32.6%), while turbid ascitic fluid was noted in 12 cases (26.1%). Inflammatory masses or nodules, described as whitish, vernix-like aggregates with surrounding inflammation on peritoneal or serosal surfaces, were found in 10 cases (21.7%). No specific pathological findings were reported in an equal number of cases (21.7%). Fibrinous material was the least common finding, seen in 4 cases (8.7%). Operative management, therefore, mainly involved peritoneal lavage and tissue sampling, with occasional omentectomy, appendectomy, drainage, adhesiolysis, or bowel resection performed, dictated by the individual findings (Table 4).

Almost every specimen submitted for pathology examination confirmed the presence of vernix elements (it was not possible to compare classic pathohistology and immunohistochemistry), while microbiological cultures were rarely positive (Table 6). Postoperatively, just over half of the women recovered uneventfully, while the rest experienced at least one complication, most often a surgical site infection (13%), intra-abdominal collection, or prolonged ileus; septic shock and other systemic problems were exceptional. Antibiotics were administered in 34 of 46 cases (73.9%). Prophylactic antibiotics were used in 25 (54.3%) cases, with documented effectiveness in 3 (12.0%) of these cases. Postoperative therapeutic antibiotics were administered in 20 cases (43.5%). It is challenging to estimate clinical effectiveness (17 of these—85.0%) (Table 6) because surgery and corticosteroids were also administered in most cases. Corticosteroids were administered in 11 of 46 cases (23.9%), with all instances showing symptom resolution and normalization of serum inflammatory markers.

Baseline C-reactive protein (CRP) levels on admission were available in 15 of 46 cases, with a median of 221 mg/L (range IQR 107–343 mg/L).

There were no maternal or neonatal deaths.

## 4. Discussion

The first description of the term VCP was by Martin S. Krumerman and Gerald J. Pouliot in 1976, after the CS [13]. VCP is characterized by an acute inflammatory response caused by the spillage of vernix caseosa, produced by the fetal sebaceous glands, into the maternal peritoneal cavity. Lanugo is a fine, soft, usually unpigmented hair that typically appears on the fetus at 16 weeks of gestation and becomes more prominent by week 20. Most fetuses shed their lanugo before birth, but it is not uncommon for newborns, especially premature babies, to be born with lanugo. This is why 84.8% of VCP pregnancies occur at term when fetuses shed their lanugo. Although previous studies and reviews claimed it is exclusive after CS, our findings indicate that 13% arise after vaginal delivery.

### 4.1. Incidence

Approximately 55 VCP cases have been reported (data were available for 46). However, the incidence of VCP is likely underestimated because many mild cases probably go unnoticed. Additionally, some VCP cases may have been misclassified as puerperal sepsis and treated with antibiotics without surgical exploration and definitive histopathology.

### 4.2. Etiopathogenesis

Although the exact mechanism of VCP remains unclear, it is generally understood that contact between fetal vernix caseosa and the maternal peritoneum and tissues triggers a granulomatous, foreign-body reaction. Granulomatous inflammation is a chronic inflammatory response of histiocytes (mature macrophages) to various infectious, autoimmune, toxic, allergic, and neoplastic conditions. In this series, it appears more like a subacute condition rather than a chronic one, occurring within 4 to 13 days after delivery (median, 8 days). This aligns with a physiological activation of histiocytes, which occurs within 24 to 48 h. Since histiocytes cannot effectively phagocytize the foreign agent, a combined innate and Th1-dominant adaptive immune response is initiated [14].

The data contradicting this concept is the application of autologous amniotic fluid in CS closure [15]. Contact with the peritoneum or intraperitoneal structures may be necessary for the reaction, or the cellular elements removed could trigger an immunologic response.

The pathophysiological explanation of VCP after vaginal delivery remains unclear. All women had previous deliveries, either cesarean section or vaginal. One possibility is that retrograde amniotic fluid flows through the Fallopian tubes after vaginal delivery or from premature rupture of membranes [7,16]. The impact of previous delivery is not clear. Tubal reflux of amniotic fluid is unlikely to occur unless there is a disruption of the natural decidual-tubal barrier or intrauterine pressure differences promote flow in this direction rather than through the cervix. In a normal gravid uterus at four or more months of gestation, intact membranes, decidual hypertrophy, stromal edema, placentation, and the cervical mucus plug typically block the cavity [16]. The induction of vaginal delivery, such as with oxytocin use [17], could increase the risk of VCP, although available data do not support this because most studies did not address it.

### 4.3. Risk Factors

Due to the limited number of cases, it is not possible to identify risk factors for VCP. Besides CS, all patients with vaginal delivery had documented previous deliveries. Approximately 80% of indications were emergent. In 95% of cases, the post-cesarean course was uneventful, with no adverse events other than those related to VCP. Factors not confirmed as risks for VCP include previous miscarriage (84.8% did not report it), the number of deliveries, and fetal sex.

In vaginal deliveries, tubal reflux could be a cause. One explanation is the high rupture of membranes (“high leak”), a type of premature rupture of membranes where the amniotic sac breaks before labor begins [16]. It occurs away from the cervix, leading to a small amount of fluid leakage. It is often mistaken for vaginal discharge or urine leakage due to the minimal fluid. If untreated, it can lead to infections. Causes include infections, cervical issues, multiple pregnancies, polyhydramnios, or previous preterm rupture of membranes or preterm labor. In such cases, tubal ligation might be protective. In that scenario, pregnancy is only possible through assisted reproductive technologies.

### 4.4. Clinical Presentation

We assume that only severe cases are published. Therefore, the clinical presentation depends on the severity of the inflammatory reaction and may go unnoticed or show mild symptoms, leading to misdiagnosis as any type of puerperal infection. A moderate inflammatory response results in vernix caseosa granuloma, while a severe reaction resembles the classic acute abdomen—peritonitis. Symptoms typically begin 4 to 13 days after delivery. There is no difference in the onset time between women who delivered vaginally and those who had a CS. Acute abdominal pain is the most common feature (89.1%), with fever over 38 °C being a common accompanying symptom (71.7%). Interestingly, a high proportion of patients present with dyspnea or tachypnea (23.9%/15.2%). These symptoms could be due to an immunologic or inflammatory (granulomatous, foreign-body type) intra-abdominal condition. Other common symptoms or signs include abdominal distension, nausea, vomiting, and constipation, which are not diagnostic but may relate to paralytic ileus caused by intra-abdominal issues (such as peritoneal nodules, inflammatory masses, or intraperitoneal collections). However, signs of ileus were described in only one patient [1].

### 4.5. Differential Diagnosis

The preoperative differential diagnoses varied. The most common was acute appendicitis (15.2%), followed by peritonitis (10.9%). VCP was initially suspected in only 4.3%. Other less frequent preoperative diagnoses included cholecystitis (4.3%), gastroenteritis, visceral injury, endometritis, intraperitoneal abscess, adnexal torsion, myofibroblastic tumor, and ruptured uterine scar—each reported in 2.2%. In 50%, the preoperative diagnosis was either unknown or not recorded.

### 4.6. Diagnosis

Achieving an accurate preoperative diagnosis is uncommon because of a nonspecific clinical presentation and the extreme rarity of this condition.

Baseline CRP levels on admission were reported in a third of cases, with a median of 221 mg/L. They remained elevated until the operation or corticosteroid treatment. This indicates that VCP is primarily a severe inflammatory condition rather than an infectious one (intraoperative swabs were positive in only 6.5% of cases).

The intraoperative or postoperative diagnoses identified VCP in 60.9%, making it the most common definitive diagnosis. Intraoperative diagnosis of acute appendicitis was established in 13.0%, and peritonitis of other or unspecified origin in 8.7%. Other findings included foreign body granuloma and bowel perforation, each observed in 2.2% of cases. In 13.0%, no conclusive diagnosis was recorded.

These findings emphasize the diagnostic challenge of VCP, which is often overlooked before surgery and frequently mimics more common intra-abdominal conditions. This may be because of the lack of consistent macroscopic features. Intraoperative descriptions most commonly mention a white, “cheesy” exudate, while adhesions or omental inflammation are also frequently seen. Turbid, bile-stained, or purulent peritoneal fluid was often found, and a smaller group of cases exhibited phlegmonous changes, walled-off intra-abdominal abscesses, macroscopic perforations, or fistulous foci. Fibrinous deposits at a perforation site are only occasionally described. In some surgeries, no specific abnormalities are observed. Interestingly, a significant decrease in adhesion formation was seen in rats treated with amniotic fluid that lacked cells and proteins [18,19].

Only histology is accurate. Histologic findings include either anucleate squamous epithelial cells, lanugo hair surrounded by keratin flakes, and an acute inflammatory reaction [7], or positive staining for Cytokeratin 1 (KL1) [20].

### 4.7. Prevention

Although evidence mainly consists of case reports and small series, most authors agree that VCP occurs after intraperitoneal spillage of amniotic fluid or vernix, leading to a foreign body granulomatosis reaction. Therefore, prevention relies on meticulous surgical technique:

- Thorough peritoneal toilet with warm normal saline before closure to remove blood clots, amniotic fluid, and visible vernix, supported by obstetric data recommending irrigation in cases with dense vernix or meconium to prevent vernix or meconium peritonitis [21].
- Careful suctioning of intrauterine contents before final uterine closure to reduce spillage [10],
- Inspection of dependent recesses (pouch of Douglas, paracolic gutters) with removal of vernix deposits [1],
- Secure hemostatic uterine closure, usually two-layered to reduce postoperative leakage [12],
- Atraumatic tissue handling and precise hemostasis to minimize inflammatory stimulus [4].

These steps are biologically plausible and explicitly recommended to prevent vernix-related peritoneal inflammation, but there are no randomized data specifically for VCP prevention. Therefore, the recommendations should be considered as best practice measures based on the mechanism and clinical literature.

### 4.8. Treatment

Due to the severe presentation of acute abdomen, extremely high levels of CRP, and uncertain but evident findings on abdominal CT or MRI, surgical exploration (93.5%) was rarely avoided. Laparotomy was the primary surgical approach, with laparoscopy used less frequently, while only 6.5% of cases avoided surgery. Most procedures were therapeutic rather than purely diagnostic. Surgery was typically performed about a week after delivery, often immediately after hospital admission.

The procedures performed, listed in decreasing order, regardless of known preoperative or intraoperative diagnosis, are a biopsy (peritoneal, omental, or mesenteric), peritoneal lavage, omentectomy, appendectomy, intraperitoneal drain placement, adhesiolysis, and bowel resection.

Antibiotics were ineffective therapeutically because VCP is not a bacterial infection but an inflammatory (granulomatous foreign body) reaction. Therefore, prophylactic antibiotics are rarely effective [20]. Most women received antibiotics, with half receiving them prophylactically and a smaller proportion therapeutically after surgery. The effectiveness of prophylactic antibiotics was limited. Although postoperative antibiotics seem effective, this could be confounded because the peritoneal cavity was irrigated, and diseased tissue or organs were removed. Removing the source could be therapeutic rather than the result of antibiotic treatment. This hypothesis is supported by the fact that only 6.5% of intraoperative microbiology tests were positive, compared to 89.1% of positive histopathology results on vernix components. Only 11 cases received corticosteroids, and most underwent surgery with peritoneal lavage as part of their management. Of these 11 cases with corticosteroid treatment, 4 were managed nonoperatively, with all patients recovering completely.

Maternal survival is 100%, but morbidity remains high. The postoperative course was uneventful for half of the patients. The surgical site infection rate was 13%, which is high, given that VCP is an inflammatory rather than an infectious condition. Since VCP develops after delivery, the neonate is unaffected. There is no fetal mortality, and birth weight is normal (3656 ± 509 g).

### 4.9. Limitations

One limitation is the 50-year period analyzed, during which imaging diagnostic technologies and immunohistochemistry improved significantly, although preoperative diagnosis remained low. Another limitation is the small number of patients, which restricts the ability to evaluate the therapeutic effect of corticosteroids on VCP. This factor could be especially important due to the inflammatory nature of the condition. Additionally, many cases do not mention all the elements and parameters that would enhance the statistical analysis.

## 5. Conclusions

Most mothers who developed VCP had term pregnancies and delivered by CS. All women with VCP after vaginal delivery had previous deliveries. Other risk factors for VCP were not detected, partly due to the small number of cases. Despite extremely low preoperative and unexpectedly low intraoperative diagnosis and treatment delay of a median of 8 days, there was no maternal or fetal mortality. The interval to symptom onset was similar between women who delivered vaginally and those who underwent CS. Abdominal pain with fever over 38 °C and dyspnea/tachypnea in the early puerperium should raise suspicion of VCP. Surgical lavage and corticosteroids are treatment options, while antibiotics have a limited role.

## Figures and Tables

**Table 1:** Baseline Characteristics (*n* = 46).

Characteristics	*n* (%)
***Maternal age*** (years)	29 ± 6 (17–43)
***Gestational age*** (at delivery)	
Term	39 (84.8)
Preterm	2 (4.3)
Unknown	5 (10.9)
***Parity***	
Nullipara	11 (23.9)
Primipara	13 (28.3)
Multipara	8 (17.4)
Unknown	14 (30.4)
***Number of pregnancies***	
0	1 (2.2)
1	7 (15.2)
2	11 (23.9)
3	5 (10.9)
6	1 (2.2)
Unknown	21 (45.7)
***Number of deliveries***	
0	9 (19.6)
1	12 (26.1)
2	7 (15.2)
3	3 (6.5)
Unknown	15 (32.6)
***Miscarriages or lost pregnancies***	
0	39 (84.8)
1	3 (6.5)
2	1 (2.2)
12	1 (2.2)
Unknown	2 (4.3)
***Maternal associated diseases***	
None	34 (72.3%)
Gestational diabetes	2 (4.3%)
Inflammatory bowel disease	2 (4.3%)
Benign gynecological conditions (leiomyomas, endometriosis)	2 (4.3%)
Bronchial asthma	1 (2.1%)
Hypothyroidism	1 (2.1%)
Diabetes mellitus, insulin-dependent	1 (2.1%)
Primary maternal toxoplasmosis	1 (2.1%)
Obstetric cholestasis	1 (2.1%)
SIADH	1 (2.1%)
Cholecystolithiasis	1 (2.1%)
***Previous deliveries***	
Cesarean	6 (13.0)
Vaginal	3 (6.5)
Not mentioned	37 (80.4)
***Current Delivery***	
Cesarean	40 (87.0)
Vaginal	6 (13.0)

**Table 2:** Obstetric characteristics and early post-cesarean outcomes.

Category/Variable	
***Indications for Cesarean section***	***n*** **(%)**
Breech presentation	8/40 (20.0)
Failure to progress in labor	11/40 (27.5)
Mechanical dystocia (*fetal/pelvic disproportion*)	5/40 (12.5)
Cord prolapse	2/40 (5.0)
Fetal compromise (*prolonged fetal bradycardia, fetal distress, pathological CTG*)	4/40 (10.0)
No specific indication recorded or elective	9/40 (22.5)
Uterine rupture	1/40 (2.5)
***Preterm rupture of membranes***	7/46 (15.2)
***Male/female infant***	
Male	8/15 (53.3)
Female	7/15 (46.7)
Unknown	31/46 (67.4)
***Weight at delivery***	*n* = 46 (in grams)
Mean ± SD	3656 ± 509 g
Min–Max	2300–4410 g
Unknown	8/46 (17.4)
***Cesarean wound healing***	*n* = 40
Normal	8/40 (20.0)
Unknown	32/40 (80.0)
N/A (vaginal delivery)	6/46 (13.0)
***Postcesarean course***	*n* = 40
Uneventful/no additional description; unknown	38/40 (95.0)
Complications *	2/40 (5.0)

* each reason appeared only once or twice, e.g., “nausea”, “pelvic or abdominal pain”, “fever”, “postpartum hemorrhage”. CTG—cardiotocography, PROM—premature rupture of membranes, SD—standard deviation, N/A—not applicable.

**Table 3:** Diagnostic findings in vernix caseosa peritonitis cases (*n* = 46).

Modality	Performed *n* (%)	Concordant ^a^ *n* (%)	Non-Concordant ^b^ *n* (%)
Abdominal/Pelvic CT	30 (65.2)	22 (73.3)	8 (26.8)
Abdominal/Pelvic Ultrasound	18 (39.1)	8 (44.4)	10 (55.6)
Chest CT	5 (10.9)	3 (60.0)	2 (40.0)
MRI	4 (8.7)	3 (75.0)	1 (25.0)
Chest X-ray	3 (6.5)	1 (33.3)	2 (66.7)
Abdominal/Pelvic X-ray	2 (4.4)	1 (50.0)	1 (50.0)
Vaginal Examination	5 (10.9)		

^a^ finding consistent with vernix-caseosa peritonitis, ^b^ finding normal or inconsistent.

**Table 4:** Surgical case overview (*n* = 46).

Variable	*n* (%)
***Preoperative differential diagnosis***	
Appendicitis	7/46 (15.2)
Peritonitis	5/46 (10.9)
Vernix caseosa peritonitis	2/46 (4.3)
Cholecystitis	2/46 (4.3)
Gastroenteritis	1/46 (2.2)
Visceral injury	1/46 (2.2)
Endometritis	1/46 (2.2)
Intraperitoneal abscess	1/46 (2.2)
Adnexal torsion	1/46 (2.2)
Myofibroblastic tumor	1/46 (2.2)
Ruptured uterine scar	1/46 (2.2)
Unknown/not recorded	23/46 (50)
***Intraoperative/Postoperative Diagnosis***	
Vernix caseosa peritonitis	28/46 (60.9)
Appendicitis	6/46 (13.0)
Peritonitis	4/46 (8.7)
Foreign body granuloma	1/46 (2.2)
Bowel perforation	1/46 (2.2)
Unknown/none recorded	6/46 (13.0)
***Intraoperative Findings***	
Turbid ascitic fluid	12/46 (26.1)
White cheesy exudates	19/46 (41.3)
Inflammatory mass or nodules	10/46 (21.7)
Fibrinous material	4/46 (8.7)
Adhesions	15/46 (32.6)
No specific finding	10/46 (21.7)
***Operating Procedures***	
Biopsy (peritoneal/omental/mesenteric)	15/46 (32.6)
Peritoneal lavage	11/46 (23.9)
Omentectomy	8/46 (17.4)
Appendectomy	7/46 (15.2)
Intraperitoneal drain placement	3/46 (6.5)
Adhesiolysis	3/46 (6.5)
Bowel resection ± anastomosis	2/46 (4.3)
Tubal ligation (patient’s request)	1/46 (2.2)
None	2/46 (4.3)

**Table 5:** Surgical treatment categories and operative timing.

Variable	*n* (%)
***Type of Operation***	
Laparotomy	31/46 (67.4)
Laparoscopy	12/46 (26.1)
No operation	3/46 (6.5)
***Surgical Management***	
Diagnostic exploration only	13/46 (28.3)
Exploration due to complications or inflammation (resection, appendectomy)	30/46 (65.2)
No operation	3/46 (6.5)
***Time from delivery to operation (days) (n = 37)***	Median 8 (IQR 4–13)
***Time from admission to operation (days) (n = 40)***	Median 0 (IQR 0–3)

**Table 6:** Perioperative outcomes and management.

	*n* (%)
***Intraoperative Microbiology/Histopathology***	
Positive microbiological findings	3/46 (6.5)
Positive pathohistological findings for VCP *	41/46 (89.1)
***Postoperative Course***	
Uneventful	24/46 (52.2)
Surgical-site infection	6/46 (13.0)
Prolonged ileus	3/46 (6.5)
Intra-abdominal abscess	5/46 (10.9)
Sepsis/hemodynamic instability	2/46 (4.3)
Other complications (e.g., respiratory, readmission)	3/46 (6.5)
Not recorded	6/46 (13.0)
***Antibiotics***	
Administered	34/46 (73.9)
Prophylactic antibiotics	25/46 (54.3)
*Effective*	3/25 (12.0)
Postoperative therapeutic antibiotics	20/46 (43.5)
*Effective*	17/20 (85.0)
***Corticosteroids***	11/46 (23.9)
*Not operated*	4/11 (36.4)
*Corticosteroids administered postoperatively*	7/11 (63.6)

* The type of histopathology or immunohistochemistry is not described in detail in most cases.

## Data Availability

No new data were created or analyzed in this study. Data sharing does not apply to this article.

## Supplementary Materials

The following supporting information can be downloaded at: https://www.mdpi.com/article/10.3390/medicina61101786/s1, Table S1: Quality assessment of case reports using JBI; Table S2: Published cases of vernix caseosa peritonitis with detailed data.

## Abbreviations

The following abbreviations are used in this manuscript:

VCP	Vernix caseosa peritonitis
CS	Cesarean section
CRP	C-reactive protein
IQR	Interquartile range
SD	Standard deviation
CTG	Cardiotocography
PROM	Premature rupture of membranes
