# Supplementary material for: Vernix Caseosa Peritonitis: A Scoping Review
Source: Medicina (Kaunas). 2025 Oct 3;61(10):1786. doi: 10.3390/medicina61101786 (PMC12566134; doi:10.3390/medicina61101786)
Supplement: Supplementary file 1 [file medicina-61-01786-s001.zip › medicina-3803146-supplementary.pdf]

Supplementary Table S1. Quality assessment of case reports using JBI.

Study ID	Q1	Q2	Q3	Q4	Q5	Q6	Q7	Q8	Overall appraisal
Abdulah, 2022	Yes	Yes	Yes	Yes	Yes	Yes	Yes	Yes	Good
Bailey, 2012	Yes	Yes	Yes	Yes	Yes	Yes	No	Yes	Good
Becker Weidman, 2020, case 1	Yes	Yes	Yes	Yes	Yes	No	Yes	Yes	Good
Becker Weidman, 2020, case 2	Yes	Yes	Yes	Yes	Yes	Yes	Yes	Yes	Good
Beorlegui, 2009	Yes	Yes	Yes	Yes	Yes	Yes	Yes	Yes	Good
Boothby, 1985	Yes	No	Yes	Yes	Yes	No	No	Yes	Fair
Cathelain, 2019	Yes	No	Yes	Yes	Yes	Yes	Yes	Yes	Good
Chambers, 2012	Yes	No	Yes	Yes	Yes	Yes	No	Yes	Good
Cummings, 2001	Yes	Yes	Yes	Yes	Yes	No	No	Yes	Good
Danawar,2 021	Yes	Yes	Yes	Yes	Yes	Yes	No	Yes	Good
Davis, 1998	Yes	Yes	Yes	Yes	No	Yes	Yes	Yes	Good
Davis, 1998	Yes	Yes	Yes	Yes	Yes	Yes	No	Yes	Good
Dhaliwal, 2023	Yes	Yes	Yes	Yes	Yes	Yes	No	Yes	Good
Freedman, 1982, case 1	Yes	Yes	Yes	Yes	Yes	No	No	Yes	Good
Freedman, 1982, case 2	Yes	No	Yes	Yes	Yes	No	No	Yes	Good
George, 1995, case 1	Yes	Yes	Yes	Yes	Yes	No	No	Yes	Good
George, 1995, case 2	Yes	Yes	Yes	Yes	Yes	Yes	Yes	Yes	Good
Hart, 2023	Yes	Yes	Yes	Yes	NA	NA	Yes	Yes	Good
Krumerman, 1976	Yes	Yes	Yes	Yes	No	No	No	Yes	Fair
Maccio, 2018	Yes	Yes	Yes	Yes	Yes	Yes	Yes	Yes	Good
Magdalenic, 2023	Yes	Yes	Yes	Yes	Yes	Yes	Yes	Yes	Good
Mahmoud, 1997, case 1	Yes	Yes	Yes	Yes	Yes	Yes	Yes	Yes	Good
Mahmoud, 1997, case 2	Yes	No	Yes	Yes	Yes	Yes	Yes	Yes	Good
Mesfer, 2024	Yes	Yes	Yes	Yes	Yes	Yes	No	Yes	Good
Mihajlovic, 2022	Yes	Yes	Yes	Yes	Yes	Yes	No	Yes	Good
Mohd Yusof, 2022	Yes	Yes	Yes	Yes	Yes	Yes	Yes	Yes	Good
Myers, 2010	Yes	Yes	Yes	Yes	Yes	Yes	Yes	Yes	Good
Nunez, 1996	Yes	Yes	Yes	Yes	Yes	Yes	Yes	Yes	Good
Sadath, 2013	Yes	No	Yes	Yes	Yes	Yes	Yes	Yes	Good
Schwartz, 1985	Yes	Yes	Yes	Yes	Yes	Yes	Yes	Yes	Good
Sellars, 2024	Yes	Yes	Yes	Yes	Yes	Yes	Yes	Yes	Good
Selo Ojeme, 2007	Yes	Yes	Yes	Yes	Yes	Yes	Yes	Yes	Good
Shoung, 2025	Yes	Yes	Yes	Yes	Yes	Yes	Yes	Yes	Good
Stuart, 2009, case 1	Yes	Yes	Yes	Yes	Yes	Yes	No	Yes	Good
Stuart, 2009, case 2	Yes	Yes	Yes	Yes	Yes	Yes	Yes	Yes	Good
Stuart, 2009, case 3	Yes	Yes	Yes	Yes	Yes	Yes	No	Yes	Good
Tawfik, 1998	Yes	Yes	Yes	Yes	Yes	Yes	Yes	Yes	Good
Val Bernal, 2014, case 1	Yes	Yes	Yes	Yes	Yes	Yes	No	Yes	Good
Val Bernal, 2014, case 2	Yes	Yes	Yes	Yes	Yes	Yes	No	Yes	Good
Vieillefosse, 2018	Yes	Yes	Yes	Yes	Yes	Yes	No	Yes	Good

Wisanto, 2010	Yes	Yes	Yes	Yes	Yes	Yes	No	Yes	Good
Yang, 2023, case 1	Yes	No	Yes	Yes	Yes	Yes	Yes	Yes	Good
Yang, 2023, case 2	Yes	Yes	Yes	Yes	Yes	Yes	No	Yes	Good
Yang, 2023, case 3	Yes	Yes	Yes	Yes	Yes	Yes	Yes	Yes	Good
Zellers, 1996, case 1	Yes	Yes	Yes	Yes	Yes	Yes	No	Yes	Good
Zellers, 1996, case 2	Yes	No	Yes	Yes	Yes	Yes	Yes	Yes	Good

Q1: Were patient's demographic characteristics clearly described?

Q2: Was the patient's history clearly described and presented as a timeline?

Q3: Was the current clinical condition of the patient on presentation clearly described?

Q4: Were diagnostic tests or assessment methods and the results clearly described?

Q5: Was the intervention(s) or treatment procedure(s) clearly described?

Q6: Was the post-intervention clinical condition clearly described?

Q7: Were adverse events (harms) or unanticipated events identified and described?

Q8: Does the case report provide takeaway lessons?

Supplementary Table S2. Case report information.

**Author**	**Title**	**DOI/PMID**
Abdulah, 2022	Vernix caseosa peritonitis following vaginal delivery: Cheesy peritonitis	10.4103/fjs.fjs_53_22
Bailey, 2012	Laparoscopic experience with vernix caseosa peritonitis	10.1007/s00464-012-2320-
Becker Weidman, 2020	Computed tomography findings in vernix caseosa peritonitis	10.1097/RCT.0000000000001079
Beorlegui, 2009	Peritonitis puerperal por vernix caseosa	10.47892/rgp.2009.291.437
Boothby, 1985	Vernix caseosa granuloma: a rare complication of cesarean section	4071157
Cathelain, 2019	Péritonite à vernix caseosa après accouchement voie basse: une complication exceptionnelle et méconnue du post-partum [Vernix caseosa peritonitis after vaginal delivery: a rare and unrecognized postpartum complication]	10.1308/003588412X13373405385296
Chambers, 2012	Delayed presentation of vernix caseosa peritonitis	10.1308/003588412x13373405385296
Cummings, 2001	Vernix caseosa peritonitis presenting post partum as acute cholecystitis	11504265
Danawar, 2021	Vernix caseosa peritonitis as a rare cause of acute abdomen after cesarean section	10.7759/cureus.17953
Davis, 1998	Vernix caseosa peritonitis: report of two cases with antenatal onset	10.1093/ajcp/109.3.320
Dhaliwal, 2023	Culture-Negative Fibrinous Peritonitis in a Postpartum Female	10.7759/cureus.43339
Freedman, 1982	Meconium granulomas in post-cesarean section patients. Obstetrics & Gynecology, 59(3):383–385.	7078887.
George, 1995	Vernix caseosa peritonitis: An infrequent complication of cesarean section with distinctive histopathologic features. American Journal of Clinical Pathology, 103(6):681–684	10.1093/ajcp/103.6.681
Hart, 2023	Vernix caseosa peritonitis: a novel case with colonic perforation	10.26635/6965.6092
Krumerman, 1976	Maternal vernix caseosa peritonitis: Rare complication of cesarean section.	1068361

Maccio, 2018	A PET-positive rapidly growing mass of the abdominal wall after cesarean section with an unexpected diagnosis of vernix caseosa granuloma: A case report	10.1186/s10397-018-1050-8
Magdalenic, 2023	Vernix caseosa peritonitis – A case report and review of the literature	10.6016/ZdravVestn.3397
Mahmoud, 1997	Vernix caseosa: An unusual cause of post-cesarean section peritonitis.	9128220
Mesfer, 2024	Vernix Caseosa Peritonitis: A case report	10.54905/disssi.v28i153.e134ms3453
Mihajlovic, 2022	Preterm Prelabour Rupture of Membranes and Vernix Caseosa Peritonitis: A Case Report	10.5937/afmnai39-31021
Mohd Yusof, 2022	A Case of Vernix Caseosa Peritonitis	10.12659/ajcr.938276
Myers, 2010	Radiology of vernix caseosa peritonitis: case report and discussion	10.1111/j.1754-9485.2011.02271.x
Nunez, 1996	Vernix caseosa peritonitis. American Journal of Clinical Pathology	10.1093/ajcp/105.5.657
Sadath, 2013	Vernix caseosa peritonitis after vaginal delivery. Clinical Medicine Insights: Case Reports, 6:147–152.	10.4137/CCRep.S12771
Schwartz, 1985	Maternal vernix caseosa peritonitis following premature rupture of fetal membranes. JAMA, 254(7):948–950.	3894706
Sellars, 2024	Vernix caseosa peritonitis: a mimic for post-partum acute appendicitis (case presentation)	10.18203/2349-2902.isj20240317
Selo Ojeme, 2007	Vernix caseosa peritonitis	10.1080/01443610701582792
Shoung, 2025	The role of medical imaging in the management of vernix caseosa peritonitis: A case report	10.1016/j.radcr.2025.01.087
Stuart, 2009	Vernix caseosa peritonitis – no longer rare or innocent: a case series	10.1186/1752-1947-3-60
Tawfik, 1998	Vernix caseosa peritonitis as a rare complication of cesarean section. A case report.	9653703
Val Bernal, 2014	Vernix caseosa peritonitis: report of two cases	10.5146/tjpath.2014.01275
Vieillefosse, 2018	A case study of vernix caseosa peritonitis	10.1016/j.jogoh.2018.08.012
Wisanto, 2010	A cheesy diagnosis	10.1016/S0140-6736(10)60875-6
Yang, 2023	Vernix Caseosa Peritonitis Causing Acute Abdomen After Cesarean Section: A Case Series	10.12659/AJCR.938276
Zellers, 1996	Complications From Cesarean Section	10.1093/ajcp/105.2.251
